# UK adaptive radiotherapy practices for head and neck cancer patients

**DOI:** 10.1259/bjro.20200051

**Published:** 2020-12-11

**Authors:** Victor Shing-Cheung LEE, Giuseppe SchettIno, Andrew Nisbet

**Affiliations:** 1 Department of Medical Physics, Royal Surrey County Hospital NHS Foundation Trust, Guildford, UK; 2 Department of Physics, University of Surrey, Guildford, UK; 3 Medical Radiation Science group, The National Physical Laboratory, Teddington, UK; 4 Department of Medical Physics & Biomedical Engineering, University College London, London, UK

## Abstract

**Objective:**

To provide evidence on the extent and manner in which adaptive practices have been employed in the UK and identify the main barriers for the clinical implementation of adaptive radiotherapy (ART) in head and neck (HN) cancer cases.

**Methods:**

In December 2019, a Supplementary Material 1, of 23 questions, was sent to all UK radiotherapy centres (67). This covered general information to current ART practices and perceived barriers to implementation.

**Results:**

31 centres responded (46%). 56% responding centres employed ART for between 10 and 20 patients/annum. 96% of respondents were using CBCT either alone or with other modalities for assessing “weight loss” and “shell gap,” which were the main reasons for ART. Adaptation usually occurs at week three or four during the radiotherapy treatment. 25 responding centres used an online image-guided radiotherapy (IGRT) approach and 20 used an offline *ad hoc* ART approach, either with or without protocol level. Nearly 70% of respondents required 2 to 3 days to create an adaptive plan and 95% used 3–5 mm adaptive planning target volume margins. All centres performed pre-treatment QA. “Limited staff resources” and “lack of clinical relevance” were identified as the two main barriers for ART implementation.

**Conclusion:**

There is no consensus in adaptive practice for HN cancer patients across the UK. For those centres not employing ART, similar clinical implementation barriers were identified.

**Advances in knowledge:**

An insight into contemporary UK practices of ART for HN cancer patients indicating national guidance for ART implementation for HN cancer patients may be required

## Introduction

Head and neck (HN) cancer is the eighth most common cancer in the UK and there are around 12 200 new cases every year.^1^ According to Cancer Research UK,^2^ between 43 and 85% of those HN cancers have radiotherapy as part of their primary cancer treatment and the incident rates have increased by fifth in the past 10 years. Radiotherapy is a crucial component of patient treatment. Advanced technology development in radiotherapy such as intensity-modulated radiotherapy (IMRT), volumetric-modulated arc therapy (VMAT), image-guided radiotherapy (IGRT) and adaptive radiotherapy (ART) has contributed to a better quality of life and reduced late toxicity for those HN cancer patients.^3–5^

An initial radiation treatment plan may not suit a patient well throughout the entire course of treatment due to post-surgical oedema, weight loss or a change in tumour size or shape. Therefore, an adaptive plan may be created by acquiring a new set of images at some point over the treatment course and applying new parameters, for example, new volumes or different prescription dose levels, for the remainder of the treatment. This process is called ART which varies across different radiotherapy centres, but the main goals are the same. First, increase the precision of treatment, so that the radiation dose to the target volumes and organs at risk (OAR) are closely matched with the original treatment plan. This first stage can be achieved with IGRT facilities for eliminating random and systematic setup errors if daily online imaging is applied. Second, alter outlines of target volumes or OARs to address shrinkage and anatomical variations in order to reduce toxicities which can be achieved by creating a new adaptive plan. Finally, alter prescription dose levels in order to increase the tumour control or ensure the OARs are still within dose tolerance.^6^

Although ART plays an increasingly important role in radiotherapy, there is a lack of international consensus on how to implement clinically. Moreover, no ART survey has been done yet across the UK for HN cancer. Common questions have been raised by many researchers, including which patients require ART and when the best time point during a treatment course is to deliver an adaptive plan. This survey aims to provide evidence on these types of questions which can be used to benchmark adaptive practices in radiotherapy centres and identify solutions to overcome any barriers for clinical implementation.

## Methods and materials

An Supplementary Material 1 was designed and sent through the UK head of radiotherapy physics network in December, 2019 and closed in February 2020, which includes 62 NHS centres and five private centres. (Note that the same technique within a private organisation but with different geographical locations is counted as a single centre within this survey). A pilot survey had been sent to three centres beforehand in order to refine the questions and a reminder email had been sent in January, 2020. The survey was performed with commercial software, Qualtrics^XM^, licensed by the University of Surrey as a secure portal for performing such surveys. Qualtrics^XM^ is much more secure than the use of open source software which has been used in other surveys.

This survey was designed to assess the followings and a blank questionnaire is included in the Supplementary Material 1.

- General information on radiotherapy centres

1. Types & number of treatment units
2. Number of HN cancer patients treated per annum
3. Main types of HN cancer
4. Number of HN cancer patients required ART

- IGRT & Adaptive strategy used for HN cancer patients

1. Imaging used for trigger ART
2. IGRT approach
3. Reasons for ART
4. ART approach & workflow (*e.g.* rescan, re-contour and re-plan),

- Quality Assurance (QA)
- Barriers & future plans for ART implementation

## Results

**Figure 1.**
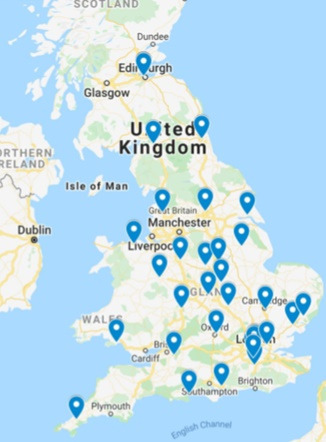
Geographical spread of 31 radiotherapy centres across the UK.

The response data were collected and analysed in February 2020. Thirty-one radiotherapy centres responded, with one radiotherapy centre excluded due to its location being outside the UK. The overall response rate was 46% (31/67), with 29 NHS radiotherapy centres and two in the private sector. The 31 radiotherapy centres demonstrate a wide geographic spread across the UK (Figure 1).

### Current status

**Figure 2.**
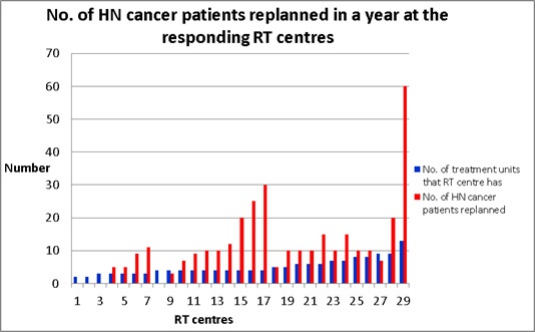
Number of treatment units in each responding RT centre and number of HN cancer patients requiring ART in a year.

90% (28/31) of responding RT centres are performing ART in the UK. However, not all 28 centres replied to all the questions. Figure 2 shows the distribution of treatment units across the participating RT centres and the majority of them have four treatment units (32%, 10/31). All centres have at least one linac. Three centres have “Tomotherapy” and “Cyberknife”, two have “Gamma knife” and “Halcyon” and one has an “MR-linac” while two respondents selected “others” (*i.e.* superficial and purchasing Halcyon). No responding centre has “Vero” or a “Proton” treatment unit.

The types of HN cancer patients who are treated are similar among all the centres. However, certain types of HN cancer patients are more common, that is, oropharyngeal cancer, tongue cancer, laryngeal cancer and “others”. The “others” have been identified as normally bulky and requiring ART.^7,8^

**Figure 3.**
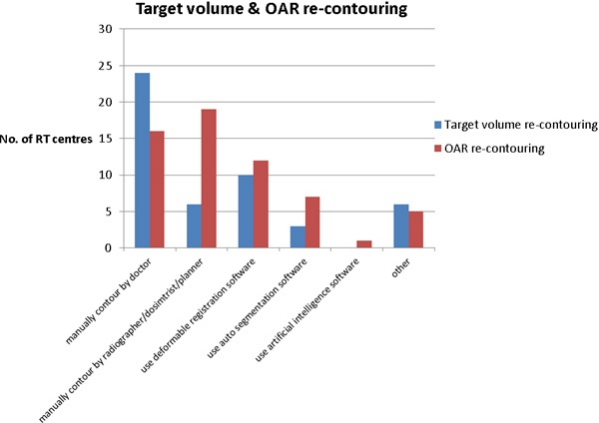
Different approaches for re-contouring target volume and OAR have been used in creating an adaptive plan.

The majority of RT centres (14/25, 56%) performing ART for HN cancer patients employ ART for between 10 and 20 patients per annum which is approximately 10% of the majority of RT centres’ HN cancer patients per annum (*i.e.*101–200 in 52%, 16/31) (Table 1). The number of HN cancer patients replanned per year across the country is shown in Table 2. However, one centre with 13 treatment units had 60 patients required for ART (Figure 2) per annum. It had more than 500 HN cancer patients per year and it had been using auto-segmentation tool for contouring both target volumes & OARs (Figure 3) which could speed up the ART process, thereby, could produce an adaptive plan in a day (Table 3).

**Table 1.** Number of HN patients approximately treated in a year in each of the responding RT centres

No. of HN patients treated per year	No. of RT centres (%)
0	1 (3%)
<50	1 (3%)
51–100	7 (23%)
101–200	16 (52%)
201–300	4 (13%)
301–400	1 (3%)
401–500	0 (0%)
>500	1 (3%)

**Table 2.** Number of HN cancer patients replanned in a year

No. of patients replanned per year	No. of RT centres (%)
<10	8/25 (32%)
≥10 to 20	14/25 (56%)
≥21 to 30	2/25 (8%)
≥31	1/25 (4%)

**Table 3.** How many days required to create an ART plan among RT centres across UK

Days required to create ART plan	No. of RT centres (%)
<1 day	0
1 day	3/26 (12%)
2 days	8/26 (31%)
3 days	10/26 (38%)
>3 days	5/26 (19%)

### Imaging & adaptive strategy

Appropriate imaging used is crucial for ART decision-making. 73% (19/26) responding RT centres used CBCT only for assessing whether ART is required and seven centres used CBCT with other modalities (kV EPID, MVCT or CT). However, no centre reported using MRI to trigger the ART process although one centre has recently installed an MR-linac. The details can be found in Table 4. The results show that CBCT plays a dominant role for assessing whether HN patients require ART.

**Table 4.** Types of imaging used to trigger the ART process

Imaging used to trigger ART	No. of RT centres
CBCT alone	19/26 (73%)
kV EPID alone	1/26 (4%)
CBCT & kV EPID	2/26 (8%)
CBCT & MVCT	2/26 (8%)
CBCT & CT	1/26 (4%)
CBCT, kV EPID & CT	1/26 (4%)
MR	0
other (please specify)	0

**Figure 4.**
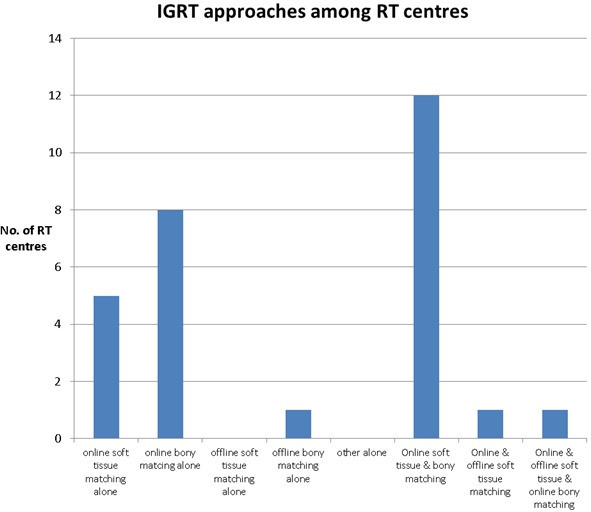
IGRT approaches have been used by the responding RT centres.

Figure 4 shows that most responding RT centres perform ART using an online approach (25 centres) with either soft tissue (five centre) or bony matching alone (eight centre) or both together (12 centres). This is aligned with IGRT guidance^9^ in order to reduce both systematic and random errors to achieve the first step of ART by adapting treatment positions. One centre used offline bony matching alone.

**Figure 5.**
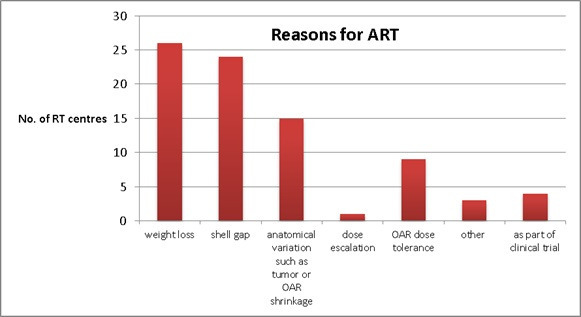
Main reasons for RT centre to perform ART for HN cancer patients.

The main reasons reported that RT centres perform ART for their HN cancer patients are weight loss (26 centres), shell gap (24 centres), anatomical variations such as tumour or OARs shrinkage (15 centres) and OAR dose tolerance (nine centres) in Figure 5. The other reasons are dose escalation (one centre), part of clinical trial (four centres) and “others” (three centres) such as “inconsistent setup”, “changes in shape resulting in failed dose constraints”, and “risk to target coverage following contour increased”.

**Figure 6.**
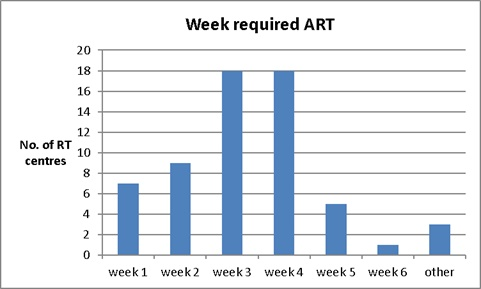
The time scale for an adaptive process to be triggered for HN cancer patients during their course of radiotherapy treatment.

One question that concerns many researchers is at what stage of treatment is ART required for HN cancer patients. Figure 6 shows that from responding centres, ART occurs at week three or 4 (18 centres) which is approximately in the middle of a 30 fraction regimen (*i.e.* from fraction 15 to fraction 25). Three centres chose “others” such as “ at any point, but mostly towards the end of treatment”, “patient specific monitored weekly and discussed at MDT” and “ at any time”.

**Figure 7.**
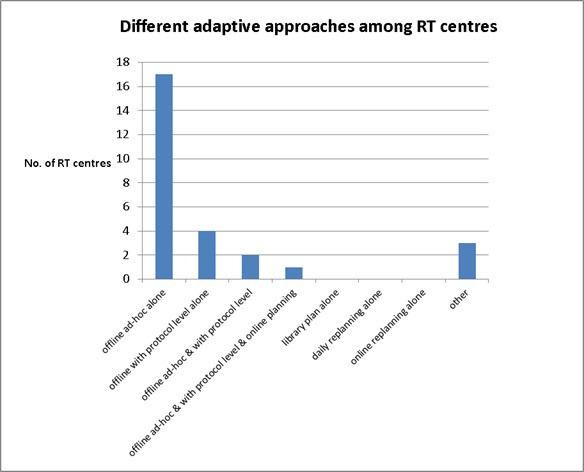
Different adaptive approaches have been used among RT centres across UK.

Due to lack of national or international adaptive guidance for action levels, 68% centres (17/25) chose to use an “offline *ad-hoc* alone” approach (Figure 7), thereby, allowing time to assess on an individual basis. One centre (1/25) selected “offline *ad-hoc* & with action level & online planning” together. No centres are using “library plan alone”, “daily replanning alone” and “online replanning alone” adaptive approaches. There were three centres which chose “others” which are likely to look at individual case by case on the dose assessment based on CBCT dose calculation on target volumes and OAR dose tolerance.

### ART planning workflow

An adaptive process normally increases the clinical workload in the department unless it is already scheduled into the routine planning pathway. Therefore, a rapid and robust ART planning workflow is vital. 100% of responding RT centres were using commercial software for performing ART, with no centre using in-house software. No responding RT centre was using a fully automated process to perform ART. The majority were using an unautomated process (22/26, 85%) while a few centres (4/26, 15%) were using semi-automated process.

Approximately, 70% (18/26) of responding RT centres required 2 or 3 days to create an adaptive plan while 20% (5/26) of them required more than 3 days (Table 3). Three centres could create an adaptive plan in a day without automated process. However, these centres all had “second independent dose calculation” as one of their pre-treatment QA procedures and with two of these centres used “deformable registration software” which could make the ART process more robust and speed up the process. There was no centre that can create an adaptive plan within a day.

A robust adaptive planning pathway should minimise one of the most time-consuming tasks during the process, which is to re-contour the target volumes and OARs. Apart from manually re-contouring for both target volumes and OARs, the “deformable registration software” had been used as the second highest option (Figure 3). There are 12 RT centres (12/28) using deformable registration tools only for contouring OARs and 10 RT centres (10/28) are using deformable registration tools for contouring both target volumes and OARs. Figure 3 shows that target volume re-contouring was carried out by a doctor predominantly (24 centres) and OARs re-contouring mostly by treatment planning staff (19 centres). More RT centres were using “auto-segmentation software” for re-contouring OARs (seven centres) rather than for target volumes (three centres) and only one centre was using “artificial intelligence software”.

Another crucial element in ART planning process is what margins should be used from adaptive clinical target volume (CTV) to planning target volume (PTV). Nearly 50% of responding RT centres (12/26, 46%) were using 3 mm margins from adaptive CTV to PTV with one centre using 2 mm margins (Table 5). Nearly 95% of centres were using 3 to 5 mm margins from adaptive CTV to PTV.

**Table 5.** The margins used for creating PTV from adaptive CTV

Margins (CTV-PTV)	No. of RT centres (%)
0 mm	0
1 mm	0
2 mm	1/26 (4%)
3 mm	12/26 (46%)
4 mm	4/26 (15%)
5 mm	9/26 (35%)
>5 mm	0
others (please specify)	0

### Quality Assurance (QA)

**Figure 8.**
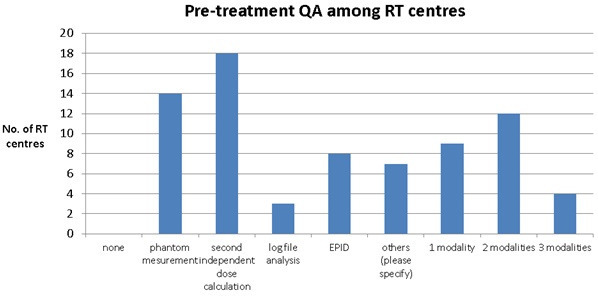
Pre-treatment QA has been used among RT centres across UK.

QA could be time-consuming or reducing treatment capacity if “phantom measurement” is performed at the treatment unit during normal working hours. 100% of RT centres were performing pre-treatment QA before delivery of the adaptive plan for the patient. “Phantom measurement” (14) and “second independent dose calculation” (18) were the top two options for RT centres (Figure 8). Most RT centres preferred to use either one modality alone (9) or two modalities together (12). However, there were only three centres using “second independent dose calculation” alone.

### Current barriers & future plans

**Figure 9.**
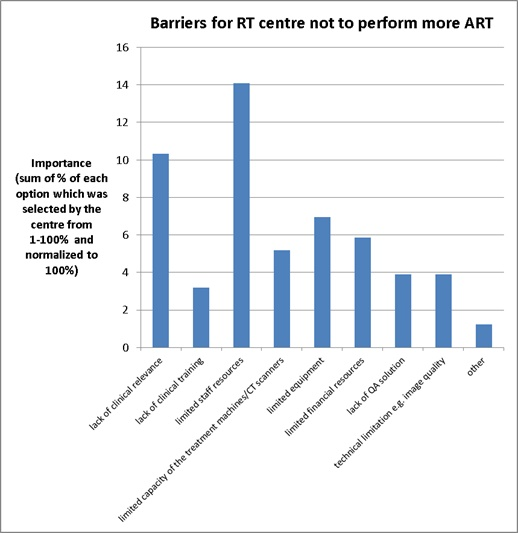
The common barriers were identified for RT centre not to perform more ART across UK.

The main barriers for RT centres to perform more ART were “limited staff resources” and “lack of clinical relevance” (Figure 9). The other barriers had all similar importance and are listed as “limited equipment”, “limited finance resources”, “limited capacity of treatment machines/CT scanners”, “lack of QA solution”, “technical limitation *e.g*. image quality” and “lack of clinical training”. Some centres selected “others” for the reason of “lack of national or international guidance, especially action level”. One centre also mentioned “limited treatment planning license” as one of their barriers.

With regard to plans for developing more ART or to consider starting ART if they were not currently doing so, 13 centres said that they have plans to develop more ART while seven centres said “No”. For the centres which currently are not doing any ART and would not consider starting ART, reasons given included “no head and neck patients in their centre” and “they assessed patients on an individual basis for any anatomical change and found they very rarely required re-planning”.

Generally, RT centres have plans if they have installed the Halcyon Ethos system (two centres), MR-linac (one centre), Precise-ART (one centre) or trying to automate the ART process (two centres) in order to perform ART directly. Some centres are trying indirect way to improve the efficiency of ART such as AI contouring software (one centre) or deformable registration software (one centre). Three centres would like to perform more ART under clinical trials. Two centres are currently in the process of reviewing the evidence base, commercial solution and auditing.

## Discussion

This survey provides an insight into the variation in current practices of ART across the UK for HN cancer. The survey included nearly 50% of RT centres in the UK with a wide geographical spread across the country. The average survey response rate from organisations is 35.7% (with standard deviation 18.8%).^10^

It is possible that RT centres not currently performing ART were less inclined to complete the ART survey. Only three centres responding indicated that they were not doing ART.

A recent survey has indicated the most common cancer site that would benefit from using ART is HN (92%), followed by lung (52%) and pelvic tumours (44%).^11^ Although they did not show the overall survey response rate and each respondent only represented an individual, it did indicate that adaptive practice is vital for HN cancer patients.

According to the survey results, more than half of the RT centres (16/31) have 101–200 HN cancer patients per annum. From Table 1, this survey accounts for approximately 3000–6000 HN cancer patients receiving radiotherapy, approximately half of the HN cancer patients in the UK who require radiotherapy according to the statistics of Cancer Research UK (between 5000 and 10 000 of 12,200 HN cancer patients required radiotherapy).^1,2^ The most common number of patients treated with ART was between 10 and 20 per annum (14/25 centres). From this snapshot, about 10% of HN cancer patients required ART. No information is available on how many patients were actually assessed for ART, which in itself may be a time-consuming activity. It has been argued that most HN cancer patients would potentially benefit from ART in terms of target volume coverage and OARs sparing^7,12,13^ although the existing clinical data on the effect of ART is still limited.

The survey results showed that 96% of respondents were using CBCT either alone or with other modalities (kV EPID, MVCT) to trigger ART across the UK. Similarly, a national survey of American Society for Radiation Oncology members, 92% of responders (total 607 responders) were using kV CBCT or MVCT for IGRT approaches.^14^ Moreover, most ART studies published in the literature are using CBCT for assessing the requirement for ART which agrees with the findings of this survey.^15–17^

One of the key elements to perform ART is the quality of imaging, which should be good enough to enable an ART decision. One RT centre with a recently installed MR-linac did not appear to be using the MR capabilities for routine clinical adaptive work at this time. It was indicated in the questionnaire that “online adaptive H&N treatments on MR-Linac will be increased in 2020. Offline scheduled adaptive CT & C-Arm based clinical trial for H&N to increase recruitment in 2020”.

Another key element of imaging quality is the ability to detect both translational and rotational errors, which CBCT does^18^ and is the best alternative to standard CT. It also provides good 3D soft tissue visualisation. In this survey, 25 responding centres used an online image-guided radiotherapy (IGRT) approach with either “online soft tissue or bony matching alone” or both together which agrees with other studies and guidance.^9,19^ This online imaging strategy is able to correct for both systematic and random errors.^9^

Weight loss, shell gaps, anatomical variations and OAR dose tolerance are the top four reasons identified in this survey for performing ART and adaptation usually occurs at week 3 or 4 (18 centres) during a 30 fractions regimen which is in agreement with the studies in the literature.^20–27^

According to our survey results, 68% of respondents (17/25) were using an “offline *ad-hoc* alone” adaptive approach. This could be because ART requires at least 2 or 3 days to create an adaptive plan (18/26 centres, 69%) and most centres indicated a “lack of staff resources” (Figure 9), indicating ART is a labour-intensive process. One of the most time-consuming tasks is to re-contour the target volumes and OARs. Our findings showed that in the majority of RT centres (24), target volumes were re-contoured by the doctors, while OARs were re-contoured by the treatment planning staff (19 centres). This survey also shows some centres (10–12) are starting to use deformable registration software or auto-segmentation (3–7 centres) for both target and OARs in the adaptive plan. However, there was only one centre using AI for re-contouring OARs. Although not every centre is currently using those technologies for re-contouring, this is one of the key elements of ART. This will become more popular or mature in the near future and help improve the efficiency of the ART process.

95% of respondents are using 3–5 mm margins for adaptive CTV to PTV with a number of centres stating this was the “same as initial planning margins” in the free text component of the questionnaire. A recent study by Navran et al^28^ showed that by reducing CTV-PTV margin from 5 to 3 mm combined with daily CBCT, it is possible to reduce radiation-related toxicity without compromising treatment outcome.^28^ Similarly, Wu et al^8^ found that CTV-PTV margin could be reduced to 0 mm without compromised target volume doses, with 22% improvement in parotid mean dose in moving from 5 to 0 mm CTV-PTV margin. In contrast to Wu et al^8^, van Kranen et al^29^ concluded that although an improvement in OAR mean dose of approximately 1 Gy/mm could be found with margins reduced from 5 to 3mm or 0 mm, 27% of all CTVs (73 CTVs in 19 patients) would lose target coverage for 0 mm margin. However, a CBCT-based ART workflow with 0 mm margin was proved to be feasible to restore CTV coverage.

On the other hand, no matter what margins are used, if the adaptive target volume is delineated incorrectly, then the dose delivered to the target volumes will be suboptimal. Therefore, it is crucial to have a peer-review process for the adaptive target volumes as recommended by the RCR guidance for all individualised volumes.^30^

Quality assurance (QA) for the adaptive plan is as important as for the original treatment plan. All centres performed pre-treatment QA although the time taken to perform these checks was not assessed. Additionally, phantom measurement could potentially occupy the treatment machine capacity if pre-treatment QA is performed during normal working hours. Second independent dose calculation was used in 18 centres, but only three centres were using it alone. This indicated that RT centres may be reluctant to only use a second independent dose calculation which could potentially speed up the QA process without imposing any impact to the treatment unit capacity.

The main barriers for performing ART were “limited staff resources” and “lack of clinical relevance”. The former is expected given ART is labour-intensive, and given shortage of therapeutic radiographers (7.0%)^31^ , physicists (9.2%) ^32^and consultant clinical oncologist (10%)^33^ in the 2019 census data. Although it may not solve the limited staff resources issue, using auto-contouring tools and automating ART process with the use of a second independent dose calculation alone for QA purposes may potentially minimise this labour-intensive process.

In addition, this survey also indicated many of the centres believed that there is limited evidence of clinical benefits for ART for HN cancer patients^34^ and also a lack of national or international guidance on ART processes. Clinical trials are being developed to provide appropriate clinical evidence and a number of RT centres have already started recruiting to such trials (*e.g.* Netherlands group, ARTFORCE trial;^35^ French group, GIRAFE trial).^36^ However, further collaborations for gathering data, sharing experience and eventually performing a large-scale randomised-clinical trial for ART HN cancer may be beneficial.

A number of centres in the survey confirmed that they would like to perform ART for clinical trials. The implementation of clinical trials has facilitated the adoption of new technology for RT centres and has led to changing clinical practices across the UK, for example, the “Fast forward trial”^37^ with five fraction breast treatment instead of standard 15 fractions which has now been adopted during the present Covid-19 situation for most RT centres in UK.

ART can be advised based on either changes in functional imaging (PET) or on physical changes (as covered in the scope of this ART survey). One of the multi-centre Phase II clinical trials of the functional imaging approach to ART “ARTFORCE” is a randomised-clinical trial for HN cancer patients who are treated with concomitant cisplatin and standard or adaptive high-dose radiotherapy (2% boosted to 84 Gy and the remaining GTV 70 Gy) using F-18-fluorodeoxyglucose position emission tomography (PET) to define GTV.^35^ A single centre French Phase II trial “GIRAFE” which is a prospective study to evaluate automatically deformed contours onto daily MVCT (*i.e.* Tomotherapy) and re-planning CT and also the time gain in the re-planning workflow.^36^ Recently, in the UK, “PATHOS” is a de-escalation ART treatment approach multi-centre Phase III clinical trial to tailor radiotherapy treatment for patients with HPV-positive oropharyngeal cancer to reduce side-effects, particularly swallowing problems, by lowering the prescribed dose to those after surgery in the intermediate risk group as a test arm and testing without Cisplatin in high-risk group.^38^ The development of ART clinical trials to provide reliable clinical evidence for the benefits of ART is to be welcomed.

This survey has indicated centres are starting to explore technology such as auto-segmentation, deformable registration and auto-contouring tools with AI and future studies may include the use of such technology to provide rapid ART or automation of the process. Once more adaptive QA results have been accumulated, RT centres may start to use offline second independent dose calculation alone in order to speed up the ART process. With the lack of guidance available, it is difficult to ensure consistent ART practice across the UK including the use of adaptive margins, actions levels to trigger ART, and how to perform ART in practice. As technologies and practices evolve and further evidence emerges similar follow up surveys may be considered.

## Conclusion

There is no consensus adaptive practice for HN cancer patients across the UK. However, centres experience similar clinical implementation barriers. This survey provides an insight into contemporary UK practices of ART for HN cancer patients and highlights the possible requirement for consensus national guidance for implementation of clinical ART for HN cancer patients.
